# Experimental demonstration on the deterministic quantum key distribution based on entangled photons

**DOI:** 10.1038/srep20962

**Published:** 2016-02-10

**Authors:** Hua Chen, Zhi-Yuan Zhou, Alaa Jabbar Jumaah Zangana, Zhen-Qiang Yin, Juan Wu, Yun-Guang Han, Shuang Wang, Hong-Wei Li, De-Yong He, Shelan Khasro Tawfeeq, Bao-Sen Shi, Guang-Can Guo, Wei Chen, Zheng-Fu Han

**Affiliations:** 1 Key Laboratory of Quantum Information, University of Science and Technology of China, Hefei 230026, China; 2 Synergetic Innovation Center of Quantum Information & Quantum Physics, University of Science and Technology of China, Hefei, Anhui 230026, China; 3 Institute of Laser for Postgraduate Studies, University of Baghdad, Baghdad, Iraq

## Abstract

As an important resource, entanglement light source has been used in developing quantum information technologies, such as quantum key distribution(QKD). There are few experiments implementing entanglement-based deterministic QKD protocols since the security of existing protocols may be compromised in lossy channels. In this work, we report on a loss-tolerant deterministic QKD experiment which follows a modified “Ping-Pong”(PP) protocol. The experiment results demonstrate for the first time that a secure deterministic QKD session can be fulfilled in a channel with an optical loss of 9 dB, based on a telecom-band entangled photon source. This exhibits a conceivable prospect of ultilizing entanglement light source in real-life fiber-based quantum communications.

With the help of quantum key distribution (QKD), two distant peers, usually named Alice and Bob, can achieve information-theoretic secure key exchange. Commonly, QKD performs the one-way protocol, in which Alice prepares then sends qubits to Bob while Bob measures the incoming qubits to decode the raw key bits. One of the most famous one-way protocols is BB84^1^, which has been successfully and widely demonstrated^2^,^3^,^4^,^5^,^6^,^7^,^8^,^9^,^10^,^11^. To exploit alternative approaches for QKD and wider research area of quantum communications, researchers have proposed the two-way protocols^12^,^13^,^14^,^15^,^16^,^17^,^18^. In such protocols, Bob prepares then sends qubits to Alice. Alice encodes classical information on the quantum states of the incoming qubits then sends them back to Bob. Finally, Bob performs measurements to decode messages.

Although the full potential of two-way QKD protocols have not been clearly revealed, some interesting and valuable features of such protocols have been presented. For example, “Ping-Pong” (PP) protocol^12^ and LM05^16^ protocol are determinstic^16^, which means no need of the basis choice reconciliation (necessary for BB84). Lu, H. *et al.* proved that some two-way deterministic QKD (DQKD) protocols are secure against detector-side-channel attacks on the backward channel^17^. Beaudry, N. J. *et al.* demonstrated that two-way DQKD protocols can outperform comparable one-way protocols^18^. For experimental implementations, the polarization fluctuations can be self-compensated by Faraday mirrors in the two-way transmissions^18^. These merits make two-way protocols worthy of further developments, both in the theory and experiments^19^,^20^,^21^,^22^.

Two-way protocols can be divided into two categories based on whether entanglement sources are used. Entanglement is a key resource in the research field of quantum information. The entanglement-based two-way protocols, such as quantum illumination (QI)^23^,^24^,^25^, may reach the goal of implementing broadband key distribution and quantum-secured direct communications (QSDC)^12^,^15^,^26^. Thus, although the two-way protocols without using entanglements have better performance in most occasions based on state-of-the-art technologies, the potential of entanglement-based two-way protocols need to be seriously explored, especially taking into account the rapid research progress of entanglement light sources^27^,^28^.

Among entanglement-based two-way protocols, the PP protocol is a pioneering and inspiring work. For the first time, it allows implementing DQKD, and applying super dense coding (SDC) to QKD makes it conceptually interesting^18^. Its deterministic property also permits the potential applications in quantum direct communication^15^ and quantum dialogue^29^. Compared to the two-way protocols without entanglements (like LM05), PP does not need techniques for drawbacks of multi-photon sources like weak coherent states. And the random number generator is not necessary at Bob’s side. However, the security of PP protocol in lossy quantum channels can not be guaranteed^30^,^31^,^32^,^33^, which is the major obstacle to apply it in real-life conditions. So far, the only experiment of PP protocol was presented in a free-space quantum channel within 2 meters^21^.

Researchers have been trying to improve the PP protocol, both in the security layer and information gain^13^,^34^,^35^,^36^. Recently, Han *et al.* proposed a modified version (abbr. MPP) of PP for QKD and proved its security against collective attacks in practical noisy and lossy channels^37^. In our work, the major contribution is to experimentally explore the feasibility of MPP over lossy fiber channels. We build the entangled photon source at degenerate wavelength of 1560 nm, using the hot PPKTP-Sagnac technique^38^,^39^,^40^,^41^,^42^. Telecom fibers together with variable attenuators are used as the quantum channel for travel photons and the storage unit for home photons. Additionally, the travel photons’ forward (Bob-Alice) and backward (Alice-Bob) channels share the single fiber link due to a polarization Sagnac interferometer at Alice’s side. With a 90° Faraday rotator, this interferometer is equivalent to a Faraday mirror when there is no encoding operation.

The experiment results verify the feasibility of MPP protocol over telecom fibers, at least in terms of the optical losses. To the best of our knowledge, the experiment is the first proof-of-principle demonstration of entanglement-based DQKD over a lossy channel. It demonstrates that MPP can find potential applications in real-life quantum communications based on existing fiber-optic networks.

## Protocol

It is necessary to give a description of MPP protocol. At first, Bob prepares N pairs of maximally polarization-entangled states 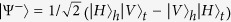, where *H* (*V*) denotes horizontally (vertically) polarized state and the subscript *h* (*t*) labels the home (travel) photon. The travel photon is sent to Alice through the forward channel (Bob-Alice), while the home photon is stored locally.

Both Alice and Bob choose message (or control) mode with probability *c* and 1 − *c* respectively. In message mode, Alice randomly applies one of four unitary encoding operations *I*_0_, *I*_1_, *Y*_0_ and *Y*_1_ (each one with probability of 1/4) to the incoming states, i.e.,


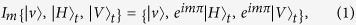



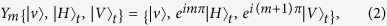


where *m* ∈ {0, 1} and 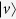 denotes the vacuum state. Note that *I*_0_ (*Y*_0_) is different from *I*_1_ (*Y*_1_) due to the existence of 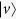. In original PP, Alice only chooses one of operations *I*_0_ and *Y*_0_. Here, adding *I*_1_ and *Y*_1_ does not affect decoding at Bob’s side, but can introduce a phase randomization to Eve’s system then limit the information that can be gained by Eve^37^. Alice records the choice of operation *I*_0_ or *I*_1_ (*Y*_0_ or *Y*_1_) as classical bit 0 (1). Then she sends the travel photon back to Bob. Under message mode, Bob performs Bell-state measurements on received photon pairs to decode messages, i.e., he records the result 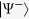 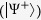 as classical bit 0 (1). In control mode, Alice (Bob) measures the travel (home) photon with projectors 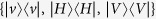.

After transmissions, Alice and Bob announce runs in message and control modes. Through sacrificing certain bits, they estimate the error rate *e* under message mode. And by sharing measurement results under control mode, they obtain probabilities *p*_*VH*_, *p*_*HH*_, *p*_*VV*_, *p*_*HV*_, *p*_*vV*_ and *p*_*vH*_, where *p*_*VH*_ means the probability that Alice receives 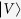 when Bob receives 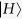, and other probabilities have similar meanings. Finally, secure key bits are generated through privacy amplification and error correction. Secure key rate *R* (bits per detection or coincidence event) is given by^37^


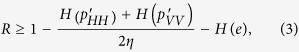


where


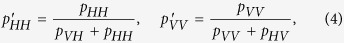


**Figure 1:**
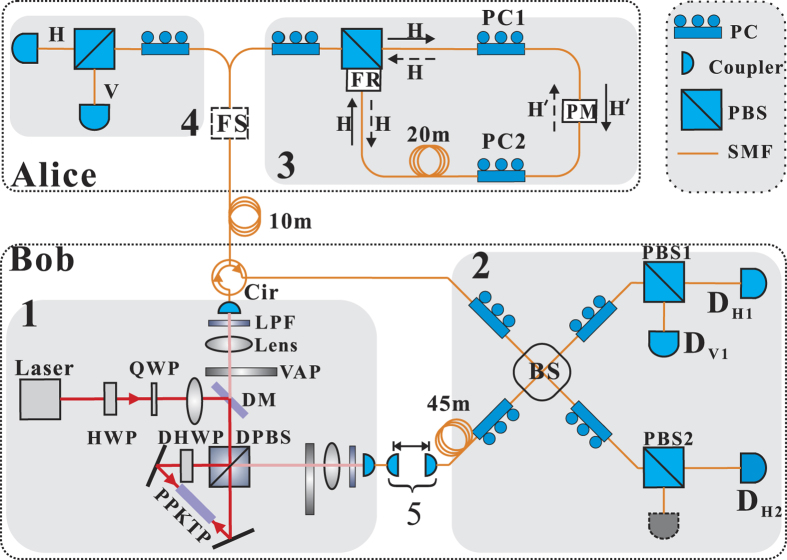
The sketch of experiment setup. Part 1–4 represent a polarization-entangled photon source, a Bell-state analyzer (BSA), a message-mode encoder and a control-mode measurement setup, respectively. Laser: 780-nm Titanium sapphire laser, Coherent MBR110; HWP: half wavelength plate; QWP: quarter wavelength plate; DM: dichroic mirror, DPBS: Dual-wavelength PBS; DHWP: Dual-wavelength HWP, 45°; PPKTP: type II periodically poled KTP, 1 mm × 2 mm × 20 mm, Raicol Crystals Ltd.; VAP: variable attenuation plates; LPF: long-pass filter; BS: beam splitter, 50:50; PC: polarization controller; SMF: single mode fiber; Cir: circulator; FS: fiber switch; PM: phase modulator; PBS: polarization beam splitter. FR: 90° Faraday rotator. PBS with FR of Alice: customized product, OZ Optics Ltd. *D*_*V*1_, *D*_*H*1_ and *D*_*H*2_: single photon detectors (SPDs). 5: the two-coupler structure.

*H*(*x*) represents the Shannon’s binary entropy function, and *η* is the transmission efficiency of the backward channel (Alice-Bob). In our setup, the forward and backward transmissions share the same fiber link, as depicted in Fig. 1. Thus, the transmission efficiency of the forward channel can also be denoted by *η*.

## Experiments setup

Firstly, we need an entangled photon source according to MPP. Due to the robustness and high brightness, the PPKTP-Sagnac configuration (cf. part 1 of Fig. 1) has become a hot technique of generating wavelength-degenerate polarization-entangled photon pairs^38^,^39^,^40^,^41^,^42^. In this work, our entangled source achieves the degenerate wavelength of 1560 nm, based on the PPKTP-Sagnac technique and continuous-wave pump.

From^38^, the output two-photon state of the polarization Sagnac interferometer (PSI) in Fig. 1 is


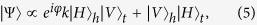


where both the ratio *k* and the phase *φ* are determined by the polarization state of the pump laser, and the subscript *h* (*t*) still labels the home (travel) photon. With the 160-mW pump power and gated InGaAs avalanche SPDs (detection efficiency: ≈10%), we obtain a coincidence count rate of about 100 cps (counts per second) and single-side count rate of 11 k cps locally. Raw visibilities under H/V and +45°/−45° bases reach 98.93% ± 0.45% and 96.03% ± 1.08% respectively. The ratio between two-pair and single-pair generation rates is less than 0.01. And the S parameter for state 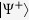 is 2.7567 ± 0.0165, which violates the Bell inequality with 46 standard deviations. More details (like the HOM dip) are given in^43^.

As shown in Fig. 1, the travel photon is sent to Alice through a 10-meter fiber channel then sent back to Bob through the same fiber. The home photon is directed into a two-coupler structure (air gap), then it is coupled into a local fiber delay line (45 m). Finally, this photon pair meet at Bob’s Bell-state analyzer (BSA), which contains a BS followed by two PBSs^44^.

**Figure 2:**
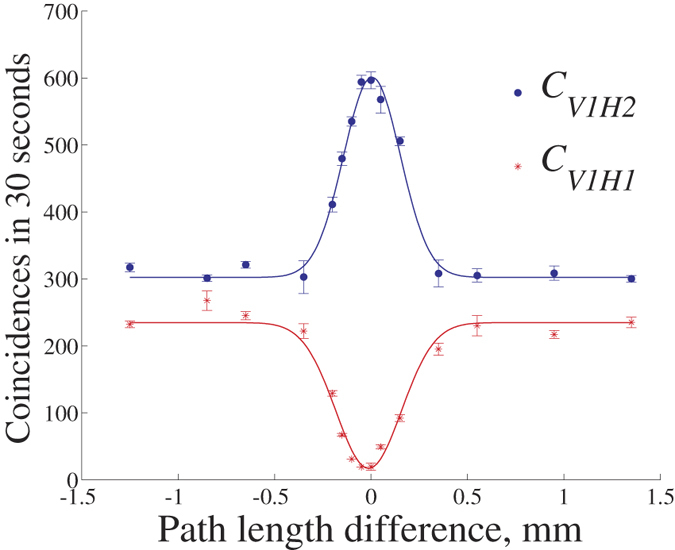
Coincidence rates *C*_*V*1*H*1_ and *C*_*V*1*H*2_ VS the difference between path lengths of the travel and home photons. The triggering rate of master SPD *D*_*V*1_ reaches 90 MHz. Notice *r* ≠ 1 when the path length difference is out of the coherence length. This is because of loss difference from the two output ports of BS of the BSA to SPDs *D*_*H*1_ and *D*_*H*2_ (including detection losses of SPDs). *D*_*V*1_ (Princeton Instruments) has 15% detection efficiency and gate width of 1 ns. *D*_*H*1_ (*D*_*H*2_), from Qasky, has detection efficiency of 8% (10%) and gate width of 2.5 ns. The dark count rates of these three detectors are 0.5, 1.2 and 1 × 10^−5^ per pulse, respectively.

To observe the two-photon interference at BSA, path lengths of the travel and home photons should be equalized within the coherence length (≈0.3 mm, as shown in Fig. 2). At first, we compensate the short path to realize the interference between two 1550-nm weak coherent pulses with the pulse width (FWHM) of 50 ps. This step can minimize the path length difference within 1 cm. Then the two-coupler structure (cf. Fig. 1) with an optical stage is used to reduce the path length difference further.

If the two-photon state coming into BSA is 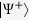 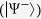, the two-photon interference may cause a coincidence count between *D*_*V*1_ and *D*_*H*1_ (*D*_*V*1_ and *D*_*H*2_). Here, we adopt master-slave type coincidence counters with 1 ns window. Electrical count signals from the master SPD *D*_*V*1_ is used to trigger the slave SPD *D*_*H*1_ (*D*_*H*2_), then electrical count signals of *D*_*H*1_ (*D*_*H*2_) are recorded to obtain the coincidence rate *C*_*V*1*H*1_ (*C*_*V*1*H*2_). For easy understanding, the travel and home photons below only refer to the ones postponed by the gated coincidence counters. In other words, our entangled source can be treated as a pulsed one with the same trigger frequency as SPD *D*_*V*1_.

As depicted in part 3 of Fig. 1, the state 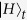 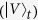, coming into Alice’s side, should be totally transmitted (reflected) by PBS into PSI of Alice, with polarization compensation of the forward channel (Bob-Alice). In this PSI, the clockwise (CW) input state of PM is adjusted by PC1 to one of its eigenstates (denoted by 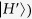. Later, we adjust PC2 to make CW propagating state reflected by PBS back into the backward channel (Alice-Bob). Correspondingly, the counterclockwise (CCW) propagating state will be transmitted by PBS back into the backward channel. And it is easy to verify that CCW input state of PM should also be 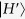. Due to the 90° FR (see Fig. 1), this PSI is functionally equivalent to a Faraday mirror, which can suppress birefringence effects and polarization-dependent loss (PDL) during two-way transmissions^45^. To avoid calibrations above, we recommend using polarization-maintaining PM and fibers.

Note that, PM is placed asymmetrically in the PSI of Alice. So, 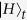 and 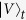 will reach PM at different times, denoted by *t*_1_ and *t*_2_ (*t*_2_ − *t*_1_ ≈ 100 ns) respectively. Ideally, Alice’s encoding operation is


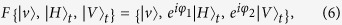


where *φ*_1_ (*φ*_2_) denotes the introduced phase on 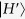 state of PM at *t*_1_ (*t*_2_). Obviously, *F* covers the four encoding operations defined by Eqs (1) and (2).

In this prototype demonstration, the control mode is run after the message mode, rather than using a switch to randomly choose message and control modes. In this mode, we replace *D*_*V*1_ with *D*_*H*2_ then connect *D*_*V*1_ to V or H ports of PBS in part 4 of Fig. 1. This setup allows measuring the travel and home photons in H/V basis at Alice’s and Bob’s sides respectively. And we record the coincidence rates *C*_*VH*_, *C*_*VV*_, *C*_*HH*_ and *C*_*HV*_. *C*_*VH*_ means the rate of coincidence counts that Alice receives 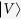 when Bob receives 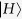, and other coincidence rates have similar meanings. From Eq. (4), we obtain


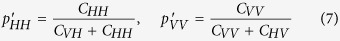


Finally, substituting 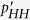, 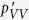, *e* and *η* into Eq. (3), we can calculate the secure key rate *R*.

## Results

Experiment results involve the two-photon interference at BSA, the encoding operation of the message mode and the loss-tolerant tests of the whole system.

In Fig. 2, we vary the path length difference to observe the destructive and full interferences. Denote the coincidence rate ratio *C*_*V*1*H*1_/*C*_*V*1*H*2_ by *r*. Ideally, *r* = 0 indicates 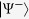, while *r* = ∞ means 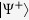. From Gaussian-fitting curves in Fig. 2, *r* reaches 0.028 under full interference. And the ratio between the value of *C*_*V*1*H*2_ under destructive interference and the one under full interference achieves 0.503. Both results show the high reliability of receiving state 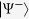 at BSA, with no Alice’ encoding operations.

In message mode, a phase modulation signal is applied to the PM at Alice’s side. When 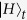 and 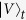 reach PM at different times, voltage levels of this signal are *V*_*D*_ + *V*_0_ and *V*_0_ respectively. Accordingly, the introduced phases on 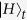 and 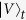 are 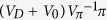 and 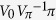 respectively, where *V*_*π*_ is the half-wave voltage (around 16.6 V). Note that the continuous-wave pumped source can not provide any synchronization information. So, we use the 1550-nm coherent pulses to synchronize the SPDs and the pulsed phase modulation voltage in advance. These 1550-nm pulses have the repetition rate of 5 MHz and pulse width (FWHM) of 50 ps.

From Eq. (6) and the initial state of 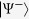, the two-photon state received by BSA becomes close to


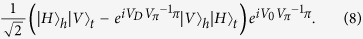


**Figure 3:**
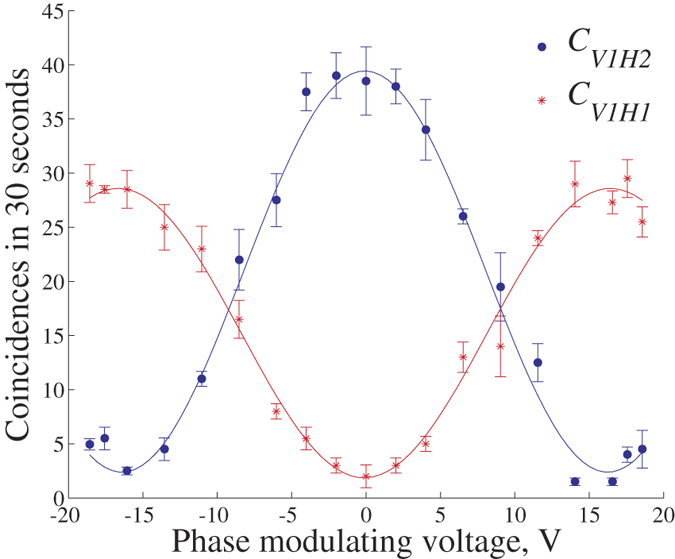
Coincidence rates *C*_*V*1*H*1_ and *C*_*V*1*H*2_ VS *V*_*D*_. When *V*_*D*_ ≠ *V*_0_, the frequency of the pulsed phase modulation signal is 5 MHz (the maximum frequency that we can provide). SPD *D*_*V*1_ is synchronized to capture the photon pairs modulated by *V*_*D*_. Thus its triggering rate is 5 MHz. The maximum of *C*_*V*1*H*1_ is smaller than the one of *C*_*V*1*H*2_, because of the loss difference mentioned in the caption of Fig. 2.

As depicted in Fig. 3, evolutions of *C*_*V*1*H*1_ and *C*_*V*1*H*2_ satisfy Eq. (8) as the voltage difference *V*_*D*_ varies. Despite the low coincidence rate and short accumulation time, the raw visibility of *C*_*V*1*H*1_ (*C*_*V*1*H*2_) achieves 88% (87%). Later, we perform the encoding operations defined by Eqs (1) and (2). Error rates *e* are listed in Table. 1. Under operations *I*_0_ and *I*_1_, the dark coincidence rate in *C*_*V*1*H*1_ is around 0.013 cps, which contributes to an error rate of about 1%. Other error rates mainly come from imperfections of the entanglement source and misalignments of polarization and optical paths during tests. Due to the loss difference after the BS of BSA, error rates under operations *I*_0_ and *I*_1_ 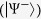 are lower than the ones under *Y*_0_ and *Y*_1_ 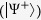, see Fig. 3 and Table. 1.

In the loss-tolerant test, we place a variable attenuator VAP into each one of the travel and home free-space channels in Part 1 of Fig. 1. Transmission efficiencies of both VAPs are adjusted to be the almost same value *ξ*. Just considering the loss, this is equivalent to make *η*, the transmission efficiency of the backward channel (Alice-Bob), become 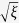. During test, the triggering rate of *D*_*V*1_ is reset to be 90 MHz for higher coincidence rates. Since 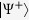 suffers a little higher error rates compared to 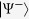 (see Table. 1), we reinitialize the two-photon state at BSA as 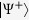 to do the worst-case analyses. And Alice’s encoding operation is fixed to *I*_1_. The run time of the message mode almost equals the one of the control mode. With the obtained error rate *e*, *η* 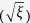, 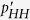 and 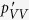, we calculate the secure key rate *R* according to Eq. (3). As long as *R* is positive, we can distill secure key from raw key bits.

**Figure 4:**
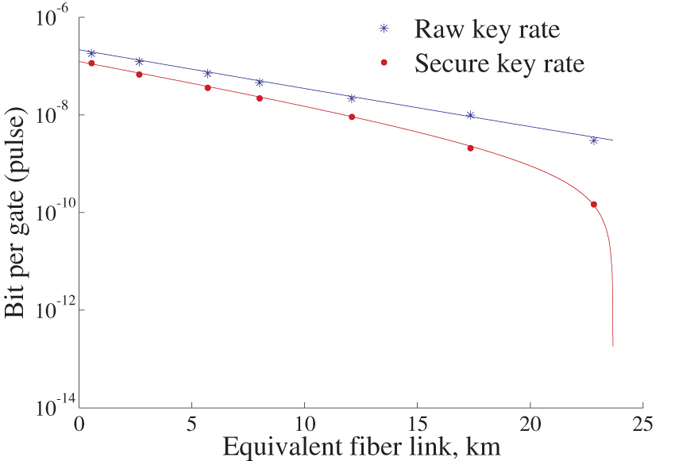
Key rates VS equivalent length of the single fiber channel. Considering a typical fiber loss of 0.2 dB/km, the equivalent fiber length equals −10 lg(*η*)/0.2 km (see values of *η* in Table 2). Secure key rate equals the product of *R* and the raw key rate. Note that *R* actually represents the bits per coincidence event. And the raw key rate means the overall coincidence rate (*C*_*V*1*H*1_ + *C*_*V*1*H*2_). Colored lines correspond to theoretical simulations based on the loss-tolerant test setup. Connecting *D*_*V*1_ and *D*_*H*1_ to the two output ports of Part 1 of Fig. 1, we find that the coincidence rate is 3.6 × 10^−6^ per coincidence window before inserting VAPs. And the average single-channel photon count rate is 3 × 10^−4^ per trigger of *D*_*V*1_. The loss of the travel (home) path connecting Part 1 and Part 2 (cf. Fig. 1) is 5.34 (1.2) dB. At the BSA of Bob, the loss of the upper (lower) optical path after BS is 1.4 (1.2) dB. Other parameters for theoretical simulations, like detection efficiencies (cf. the caption of Fig. 2), are given above.

Loss-tolerant test results are listed in Table 2. As depicted in Fig. 4, these results match theoretical simulations very well. And it is indicated that, when *R* reaches 0, the equivalent length of the fiber link between Alice and Bob can reach up to 24 km. This shows the feasibility of MPP on fiber transmissions of a few kilometers. The secure key rate (bits per second) is strongly limited by the low raw key rate (coincidences per second). With brighter high-quality entangled photon source, devices with lower losses and better detection methods, secure key rates and transmission distances can be significantly increased.

## Conclusion

Among two-way QKD protocols, original PP protocol is a seminal work, but its applications in real-life world are limited due to its insecurity in lossy channel^12^,^13^,^14^,^15^,^16^,^17^,^18^. Han *et al.* modified PP protocol through adding two extra unitary encoding operations^37^. This novel and feasible modification applies a phase randomization to Eve’s accessed system then cause the decoherence of this system. The loss-tolerant property of MPP motivates us to test it over practical lossy channels.

Actually, in both PP and MPP, the telecom fiber is a natural choice of storing home photons for a long time due to its space saving, low loss and economy. And its advantage of space saving over the free-space channel will become more important once the secure zone of Bob is limited. For future long-haul communications, we decide to realize MPP on telecom fiber. However, Bell-state analyses through interferences require travel and home photons be wavelength degenerate. So, the first obstacle that we face is to create high-bright entangled photon source at degenerate wavelengths within telecom band. Based on the outstanding PPKTP-PSI scheme, and through careful adjustment and fine temperature control (with stability of 2 mK), we successfully build the high-quality polarization-entangled photon source at degenerate wavelength of 1560 nm.

Another obstacle comes from birefringence effects and PDL during fiber transmissions, which may render the long-haul transmission of polarization-entangled states impossible. As described above, we design a PSI at Alice’s side. The PSI splits, flips then recombines orthogonal states of travel photons, and the PM is placed asymmetrically in PSI to realise multiple encoding operations conveniently. Once Alice compensates the polarization drift from forward transmission (Bob-Alice), we can avoid birefringence effects and PDL during forward and backward transmissions. In a word, our PM-PSI has merits of simplicity and robustness. It may inspire more novel designs for local operations on entangled photon pairs.

Results show the feasibility of MPP over a few kilometers fiber communications. This may find potential applications in inner-city QKD fiber networks. Compared to previous works, our experiment means a meaningful step towards the real-world applications using entanglement-based DQKD. For the future work, the potential of the PP type protocols should be continuously tapped from both physical layers and security layers. More inspirations can be obtained from these methods^13^,^34^,^35^,^36^ mentioned above to improve the capacity, information gain and security. We believe that our work will offer helpful references for the two-way QKD protocols and encourage deterministic entanglement-based quantum communications.

## Additional Information

**How to cite this article**: Chen, H. *et al.* Experimental demonstration on the deterministic quantum key distribution based on entangled photons. *Sci. Rep.* **6**, 20962; doi: 10.1038/srep20962 (2016).

## Figures and Tables

**Table 1:** Encoding operations VS settings of *V*_0_ and *V*_*D*_.

*V*_*D*_ + *V*_0_	*V*_0_	Operation	Error rate(avg.)
0	0	*I*_0_	4.1%
*V*_*π*_	*V*_*π*_	*I*_1_	4.0%
0	*V*_*π*_	*Y*_0_	5.4%
*V*_*π*_	0	*Y*_1_	5.6%

For each operation, the error rate is calculated per hour.

**Table 2:** *e*, 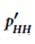 and 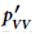 are obtained from coincidence counts accumulated within 8 minutes.

*ξ*	0.950	0.782	0.591	0.478	0.328	0.202	0.122
equivalent *η*	0.975	0.884	0.769	0.692	0.573	0.450	0.350
e(%)	2.50	3.80	3.92	4.09	4.05	6.80	7.80
*R*	0.632	0.547	0.509	0.472	0.416	0.209	0.049

Since 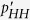 and 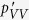 are within 2.74% ~ 3% when *ξ* ≥ 0.1, both 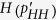 and 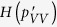 change a little. So, we set 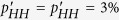 when estimating *R* (bits per coincidence event).
